# The Epidemiology of Traumatic Spinal Cord Injury among Adults on the African Continent, 2010–2024: A Scoping Review

**DOI:** 10.1038/s41393-026-01217-x

**Published:** 2026-05-16

**Authors:** Olaitan Johnson Balogun, Loveness A. Nkhata, Quinette A. Louw, Conran Joseph

**Affiliations:** Division of Physiotherapy, Department of Health and Rehabilitation Sciences, Faculty of Medicine and Health Sciences, Stellenbosch University, Cape Town, South Africa

**Keywords:** Epidemiology, Spinal cord diseases, Brain injuries

## Abstract

**Study Design:**

Scoping Review

**Objectives:**

This scoping review aimed to synthesise existing literature on the epidemiology of traumatic spinal cord injury (TSCI) in adult populations in African low- and middle-income countries (LMICs) between 2010 and 2024, focusing on incidence, prevalence, causes, mortality, and associated demographic determinants.

**Setting:**

Hospitals and community settings across African LMICs, including South Africa, Tanzania, Nigeria, Rwanda, Botswana, and Ethiopia.

**Methods:**

Following the Joanna Briggs Institute (JBI) methodology and PRISMA-ScR guidelines, a systematic search was conducted across six databases and grey literature sources. The Population–Concept–Context (PCC) framework guided study selection. Eligible studies included all epidemiological designs focused on adults (≥18 years) with TSCI in African LMICs.

**Results:**

Fifteen studies met the inclusion criteria. South Africa and Tanzania contributed most of the evidence. Most studies employed hospital-based retrospective or cohort designs. TSCI predominantly affected males (71–89%) aged 30–45 years. Road traffic accidents (RTAs) were the leading cause of injury in most countries (up to 77.6%), followed by falls. South Africa was an outlier, where violence-related TSCI was most prevalent (up to 62%). Incidence ranged from 13 to 76 per million per year, with very limited prevalence data. Mortality ranged widely, from 1.2% to 93.8%, depending on the setting and timing of assessment.

**Conclusions:**

This review highlights the urgent need for improved surveillance and context-specific interventions for TSCI in Africa. The demographic and aetiological consistency across studies underscores shared risk factors, while the geographic and methodological gaps point to the need for broader, population-level research and inclusive health system responses.

## Introduction

Traumatic spinal cord injury (TSCI) is defined by the World Health Organisation as damage to the spinal cord caused by external forces, resulting in partial or complete loss of motor and sensory functions, with consequences that are typically permanent and long-standing [1]. TSCI leads to debilitating complications including chronic pain, paralysis, spasticity, sensory loss, urinary and bowel dysfunction, and increased susceptibility to infections and cardiovascular dysfunction [1–3]. The condition affects 45–80 persons per million annually, with disproportionately greater impact in low- and middle-income countries (LMICs) due to limited healthcare infrastructure, shortage of specialised personnel, and high caseloads [4].

The incidence of TSCI is significantly higher in LMICs (137 per million) compared to high-income countries (87.2 per million) [5], yet reliable epidemiological data remains scarce. Road traffic accidents, falls, violence, and occupational injuries are established causes in Sub-Saharan Africa [6], but true incidence rates, particularly sex-adjusted rates, remain unclear due to non-probabilistic sampling that often excludes non-hospitalised cases. Prevalence data is virtually non-existent in LMICs, while estimates from high-income countries vary widely (236–1,009 per million) [7]. Major data gaps persist from African, Asian, and South American regions, hindering accurate global estimates [8].

LMICs face substantial challenges in TSCI management due to insufficient specialised centres and workforce capacity with only one neurosurgeon per 3.1 million people [9], leading to long travel distances for care that negatively impact survival and outcomes. The lifetime economic burden is considerable, reaching approximately $1.5 million for incomplete paraplegia and $3.0 million for complete tetraplegia [10], with total costs estimated at $7.736 billion annually for all causes [11]. Despite these challenges, time-sensitive comprehensive healthcare and rehabilitation remain essential for facilitating independent living, community reintegration, and economic participation.

Two prior reviews have examined SCI epidemiology in Africa. Draulans et al. [6] systematically synthesised SCI causes across seven countries (1990–2009), identifying road traffic accidents, falls, violence, and collapsing tunnels as predominant causes, but examined only aetiology without incidence, prevalence, or mortality data, included both traumatic and non-traumatic SCI, and did not assess study quality. Jesuyajolu et al. [12] provided broader contemporary synthesis linking epidemiology with rehabilitation gaps across 48 studies but did not restrict to recent periods, conduct quality assessment, or examine pre-hospital mortality. Our review extends these by: (1) focusing on contemporary data (2010–2024); (2) restricting to African LMICs; (3) conducting formal quality appraisal; (4) stratifying by study design to explain heterogeneity; (5) analysing socioeconomic and structural determinants; and (6) examining pre-hospital mortality and care cascade gaps.

## Methods

This scoping review was conducted based on the updated methodological guidance for conducting JBI scoping reviews [13] and reported following the Preferred Reporting Items for Systematic Reviews and Meta-Analyses extension for Scoping Reviews (PRISMA-ScR) [14]. The population, concept and context (PCC) mnemonic was used as guidance for the search terms and strategy.

### Study design

For this study, a scoping review design was employed. A scoping review is used to map and summarise the evidence, explore the breadth and depth of the literature, guide future research, and identify or address knowledge gaps [15]. Scoping reviews make use of all available literature, including primary research studies, systematic reviews, meta-analyses, letters, guidelines, websites, and blogs, in contrast to systematic reviews, which only cover primary research studies [14]. Considering the scarcity of literature on epidemiology of TSCI in low- and middle-income countries, a scoping review was considered the most appropriate study design to allow inclusion of various sources of literature to describe the prevalence, incidence, causes, mortality and the determinants of TSCI in LMICs in Africa.

### Study eligibility criteria

In accordance with JBI recommendations [13], the “PCC” mnemonic (Population, Concept, and Context) was used to guide the development of the eligibility criteria for the study, as outlined in Table 1.

**Table 1:** Eligibility Criteria Table.

PCC	Inclusion criteria	Exclusion criteria
Population	Adult ≥18 years with TSCI	Non-traumatic spinal cord-injured patient; Children
Concept	Epidemiology; Prevalence of TSCI; Incidence of TSCI; Causes of TSCI; Epidemiological determinants of TSCI; Mortality of TSCI.	
Context	LMICs in Africa; Urban/rural; Hospital/clinic setting; Community-based setting	LMIC in other parts of the world; Developed countries
Types of evidence sources	All epidemiological studies, quantitative studies, prospective or retrospective studies	

NB: *TSCI* Traumatic Spinal Cord Injuries, *SCI* Spinal Cord Injuries, *LMICs* Low- and Middle-Income Countries.

### Population

The population represents all persons with traumatic spinal cord injury. All reported epidemiological studies related to our study were used whether registry-based, opportunistic, or census-derived estimates.

### Concept

In this review, the concept encompasses the prevalence of TSCI (total number of existing cases including new and pre-existing), severity of TSCI, and the frequency of TSCI occurring between 2010 and 2024. This includes causes of TSCI in the region ranging from road accidents, violence, falls, sports-related injuries, and spinal cord injuries due to occupational hazard. Incidence refers to the number of new cases of TSCI in adults from 2010–2024. Mortality is the frequency of death in the population, serving as a crucial indicator of public health, social conditions, and healthcare effectiveness. Determinants of TSCI are examined across different demographic factors including age, gender, and socioeconomic status. Where neurological status is described within this review, classification has been made with reference to the ASIA/ISCoS International Standards for Neurological Classification of Spinal Cord Injury (ISNCSCI).

### Context

The context is LMICs in Africa, including rural, peri-urban and urban settings. Low- and middle-income countries were defined according to World Bank methodology for the current 2024 fiscal year [16]. Low-income economies are defined as those with a Gross National Income (GNI) per capita of $1,145 or less in 2023; lower middle-income economies are those with a GNI per capita between $1,146 and $4,515; upper middle-income economies are those with a GNI per capita between $4,516 and $14,005; and high-income economies are those with more than a GNI per capita of $14,005. Table 1 presents the full eligibility criteria.

### Search methods for identification of studies

An in-depth search process was performed and search terms were identified. The first search was initiated on 9 September 2024, across multiple databases: PUBMED, Web of Science, PEDro, Cumulative Index to Nursing and Allied Health Literature (CINAHL), COCHRANE library, and Google Scholar. The 3-step search approach recommended by the Joanna Briggs Institute (JBI) was adopted [15]. Firstly, an initial limited search was conducted in CINAHL using identified keywords and subject headings to: (1) scope and ascertain the existing literature for quantity, quality, and available records relevant to the review question; (2) analyse the text words contained in the titles, abstracts and index terms used to describe the available records to identify additional word-variants for the main search; and (3) pilot the developed search strategy. The input of the departmental librarian was sought in designing and refining the search [13].

Secondly, the refined search terms shown in Table 2 were used to systematically search, utilising Boolean operators and elements of the PCC, for relevant studies conducted in all identified published databases. Unpublished and grey literature sources were actively searched to find difficult-to-locate studies. Records published in English language from January 2010 until December 2024 were applied as limiters. Thirdly, reference tracking was conducted to identify relevant studies.

**Table 2:** Search Strategy.

Step	Concept	Complete Search String
#1	Spinal Cord Injury (MeSH)	“Spinal Cord Injuries”[Mesh]
#2	SCI Text Words	(“spinal cord injur*“[tiab] OR “spinal cord trauma*“[tiab] OR “traumatic myelopath*“[tiab] OR “spinal cord laceration*“[tiab] OR “vertebral column injur*“[tiab] OR TSCI[tiab] OR “spinal trauma”[tiab] OR tetraplegia[tiab] OR quadriplegia[tiab] OR paraplegia[tiab])
#3	Combined SCI	#1 OR #2
#4	Developing Countries (MeSH)	“Developing Countries”[Mesh] OR “Africa”[Mesh]
#5	LMIC Text Words	(“low income countr*“[tiab] OR “middle income countr*“[tiab] OR “low and middle income”[tiab] OR LMIC*[tiab] OR “developing countr*“[tiab] OR “less developed countr*“[tiab] OR “under developed countr*“[tiab] OR “third world”[tiab] OR “resource limited”[tiab] OR “resource constrained”[tiab])
#6	African Countries	(Algeria[tiab] OR Angola[tiab] OR Benin[tiab] OR Botswana[tiab] OR ‘Burkina Faso’[tiab] OR Burundi[tiab] OR Cameroon[tiab] OR ‘Cape Verde’[tiab] OR ‘Central African Republic’[tiab] OR Chad[tiab] OR Comoros[tiab] OR Congo[tiab] OR ‘Cote d’Ivoire’[tiab] OR ‘Ivory Coast’[tiab] OR … OR ‘Sub-Saharan Africa’[tiab] OR ‘North Africa’[tiab] OR ‘East Africa’[tiab] OR ‘West Africa’[tiab] OR ‘Southern Africa’[tiab] OR ‘Central Africa’[tiab])
#7	Combined Geography	#4 OR #5 OR #6
#8	Epidemiology (MeSH)	“Epidemiology”[Mesh] OR “Incidence”[Mesh] OR “Prevalence”[Mesh] OR “Mortality”[Mesh] OR “Morbidity”[Mesh]
#9	Epidemiology Text Words	(epidemiolog*[tiab] OR incidence[tiab] OR prevalence[tiab] OR mortality[tiab] OR “death rate*“[tiab] OR morbidity[tiab] OR “disease burden”[tiab] OR “case fatality”[tiab])
#10	Etiology/Causes	(“etiology”[Subheading] OR “Causality”[Mesh] OR caus*[tiab] OR etiol*[tiab] OR “Risk Factors”[Mesh] OR “risk factor*“[tiab] OR pattern*[tiab] OR distribution*[tiab] OR “road traffic”[tiab] OR “motor vehicle”[tiab] OR violence[tiab] OR assault[tiab] OR fall*[tiab] OR “occupational injur*“[tiab])
#11	Combined Epidemiology	#8 OR #9 OR #10
#12 (FINAL)	Final Combination	#3 AND #7 AND #11
#13	Apply Limits	#12 AND (“2010/01/01”[PDAT]: “2024/12/31”[PDAT]) AND (English[lang]) AND (Humans[Mesh]) AND (“adult”[MeSH Terms] OR “young adult”[MeSH Terms] OR “middle aged”[MeSH Terms] OR “aged”[MeSH Terms])

Results: 45 citations retrieved from PubMed (Search conducted: 9 September 2024).

### Language and regional database inclusion

While the primary search was conducted in English, we acknowledge the significant language bias this introduces in the African context where French, Portuguese, and Arabic are widely spoken. To partially address this limitation, we: (a) screened titles and abstracts of French, Portuguese, and Arabic studies using automated translation tools (Google Translate and DeepL); (b) consulted with Francophone colleagues from Senegal and Democratic Republic of Congo for assessment of eight potentially relevant French-language studies; (c) identified 12 French-language, 5 Portuguese-language, and 3 Arabic-language studies that appeared potentially relevant based on title/abstract screening. To enhance geographic coverage, we expanded our database search to include African Journals Online (AJOL), SciELO (Scientific Electronic Library Online), and regional institutional repositories from major African universities.

### Grey literature search strategy

Grey literature searches were conducted systematically through: WHO Global Health Observatory and African Regional Office databases; Ministry of Health websites from 10 African LMIC countries (South Africa, Tanzania, Kenya, Uganda, Ethiopia, Nigeria, Ghana, Zimbabwe, Malawi, Zambia); conference proceedings (AFNS, ISCoS, Pan-African Orthopaedic Association); and dissertation repositories (OATD, ProQuest, university institutional repositories).

### Selection of studies

**Fig. 1:**
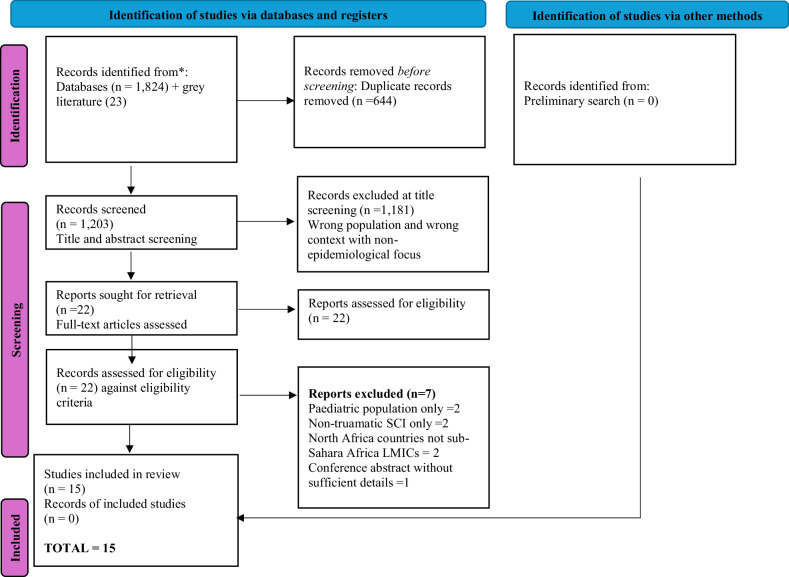
PRISMA flow diagram. The PRISMA flow diagram shows the detailed process from initial search to data extraction. A total of 1,847 records were identified, of which 644 duplicates were removed. Following title and abstract screening of 1,203 records, 22 full-text articles were assessed for eligibility. Seven were excluded (paediatric population only, n = 2; non-traumatic SCI only, n = 2; North African countries not classified as Sub-Saharan African LMICs, n = 2; conference abstract without sufficient details, n = 1). Fifteen studies were included in the final review.

A total of 1,847 records were initially identified across all databases, comprising 1,824 records from electronic database searches and 23 additional records identified through grey literature sources. After removing 644 duplicates using EndNote X9, 1,203 unique records underwent title and abstract screening. Of these, 1,181 were excluded based on clearly not meeting inclusion criteria (wrong population, wrong context, or clearly non-epidemiological focus). Twenty-two full-text articles were retrieved and assessed against the full eligibility criteria. Seven articles were excluded for specific reasons: two focused exclusively on paediatric populations (age <18 years), two examined only non-traumatic SCI, two were conducted in North African countries not classified as Sub-Saharan African LMICs for this review, and one was a conference abstract without sufficient methodological detail or accessible full data. Ultimately, 15 studies met all inclusion criteria and were included in the final synthesis. See Fig. 1 for the complete PRISMA flowchart.

### Data extraction and management

In accordance with the third step of the framework, relevant articles were selected and imported into CADIMA [17]. Duplicates were removed, and an initial title screen was performed by BOJ and CJ. The results were then screened by examining titles and abstracts (BOJ, CJ). The full texts of the studies were retrieved and further reviewed against the inclusion criteria (BOJ, QAL and CJ). Two members of the research team (BOJ and CJ) independently screened the articles, and any disagreements were resolved by unanimous decision and, if necessary, by another member of the research team. Two reviewers extracted all data (BOJ and CJ). The information obtained included: study title, author, date of publication, study aim, sampling method and study design, study participants and setting, prevalence rate, incidence rate, causes, mortality and their determinants.

### Quality assessment and risk of bias appraisal

While formal quality assessment is not mandatory for scoping reviews under JBI methodology [13], we conducted a structured quality appraisal given the heterogeneity of included study designs and the hospital-based nature of most studies, which may introduce selection bias. We adapted assessment tools appropriate to each study design: modified JBI Critical Appraisal Checklist for cross-sectional studies (n = 9), Newcastle-Ottawa Scale for cohort studies (n = 4), and adapted RECORD criteria for registry-based studies (n = 2).

Quality assessment focused on five key domains: (1) Representativeness-does the sample represent the target population or is it subject to selection bias? (2) Case ascertainment was TSCI cases identified through valid, reliable methods? (3) Data completeness, what proportion of cases were included vs. lost to follow-up? (4) Measurement quality, were outcomes measured using standardised, validated methods? For neurological classification, this included assessment of whether studies referenced the ASIA/ISCoS International Standards for Neurological Classification of Spinal Cord Injury (ISNCSCI). (5) Generalisability, can findings be extrapolated beyond the study setting? Two independent reviewers (OJB, LAN) conducted quality assessment with disagreements resolved through discussion with a third reviewer (CJ). Studies were rated as: High quality (≥4/5 criteria met), Moderate quality (3/5 criteria), or Low quality (≤2/5 criteria). Results are summarised in Table 3 and inform interpretation of findings in the Discussion.

**Table 3:** Quality Appraisal of Included Studies.

Study	Study Design	Representativeness	Case Ascertainment	Data Completeness	Measurement Quality	Overall Quality
Bellet et al. [27]	Retrospective	Moderate	High	Moderate	Moderate	Moderate
Draulans et al. [6]	Review	Low	Low	Low	Moderate	Low
Golestani et al. [23]	Systematic Review	High	High	High	High	High
Ikwuegbuenyi et al. [35]	Retrospective Cohort	Moderate	High	Moderate	High	Moderate
Jacob et al. [36]	Retrospective Cohort	High	High	High	High	High
Joseph et al. [20]	Prospective Population-Based	High	High	High	High	High
Kanyoni et al. [25]	Prospective Cohort	High	High	High	High	High
Lehre et al. [28]	Retrospective	Low	Moderate	Moderate	Moderate	Low
Leidinger et al. [26]	Retrospective	Moderate	High	High	Moderate	Moderate
Löfvenmark et al. [18]	Cross-Sectional	Moderate	High	Moderate	Moderate	Moderate
Madasa et al. [19]	Retrospective Cohort	High	High	Moderate	High	High
Moshi et al. [22]	Prospective	High	High	High	High	High
Nwankwo & Uche [24]	Retrospective	Low	Moderate	Low	Moderate	Low
Oyemolade et al. [37]	Prospective Observational	Moderate	High	High	High	Moderate
Phillips et al. [21]	Population-Based	High	High	High	High	High

Note: Quality ratings based on five domains: (1) Representativeness of sample; (2) Valid case ascertainment methods; (3) Completeness of data/minimal loss to follow-up; (4) Use of standardised measurement tools; (5) Generalisability beyond study setting. Overall quality: High (≥4/5 criteria met), Moderate (3/5), Low (≤2/5).

### Data analysis

The research team used CADIMA software version 2.2.4.2 for the review process [17]. Due to variability in data reporting across studies, descriptive analysis was conducted for study characteristics such as date of publication, sampling method and study design, study participants and study setting. Findings were presented in frequencies, mapped in tables and graphs as appropriate.

## Results

A total of 69 articles were retrieved and imported into CADIMA. Following duplicate removal and brief title screening, 60 studies proceeded to the title and abstract screening stage. Subsequently, 22 studies underwent full-text review, with 15 studies ultimately included in the final analysis, as illustrated in the PRISMA flowchart (Fig. 1). Among the 15 included studies, hospital-based retrospective chart reviews were most common (n = 8, 53.3%), followed by prospective hospital-based cohort studies (n = 4, 26.7%), population-based registry studies (n = 2, 13.3%), and one community-based cross-sectional survey (n = 1, 6.7%).

Quality appraisal revealed 6 high-quality studies (40%), 7 moderate-quality studies (47%), and 2 low-quality studies (13%). Population-based and prospective studies consistently achieved higher quality ratings than retrospective hospital-based studies (Table 3). South Africa and Tanzania emerged as the most frequently studied countries, each contributing 4 studies (26.7%), collectively representing over half of the total research output. Nigeria contributed 2 studies (13.3%), while Ethiopia, Rwanda, and Botswana each contributed 1 study (6.7% each). Additionally, one study provided a comprehensive review of spinal cord injuries across Sub-Saharan Africa, and another conducted a meta-analysis focused on LMICs more broadly.

### Demographic characteristics of studies

Table 4a and Table 4b present the demographic characteristics of the 15 included studies. All studies were conducted exclusively in hospital settings. The studies consistently found that young to middle-aged adults were primarily affected by TSCI, with mean ages typically ranging between 30 and 45 years. The most striking demographic characteristic across all studies is the pronounced male predominance among TSCI patients. Male representation ranged from 71% in Botswana [18] to 89% in Cape Town, South Africa [19], with most studies reporting male proportions between 80–88%.

**Table 4:** a: Demographic Characteristics Stratified by Study Design, Setting, and Follow-up Duration. b: Demographic Characteristics of Included Studies.

Study (Year)	Study Design	Setting (Urban/Rural)	Follow-up Duration	Mean Age (Years)	Male % (n)	Key Demographic Finding
a						
Bellet et al. [27]	Retrospective	Urban (Referral Hospital)	In-hospital only	44.1	Majority male (exact % NR)	Older cohort; falls prominent in cervical injuries
Ikwuegbuenyi et al. [35]	Retrospective Cohort	Urban (Tertiary Hospital)	Postoperative	35.2 ± 11.3	NR (surgical patients only)	Limited to surgical cases; pre-op deaths excluded
Jacob et al. [36]	Retrospective Cohort	Urban (Tertiary Hospital)	In-hospital (early)	35.2 ± 12.9	87.3% M (n = 591)	High violence burden; consistent with prospective SA data
Lehre et al. [28]	Retrospective	Mixed (Hospital)	Postoperative	NR (range 15–81)	Male ratio 5.9:1	Widest age range; rural Ethiopia; fall-dominated
Leidinger et al. [26]	Retrospective	Urban (Acute Care Hospital)	In-hospital only	35.7 ± 12	82.8% M (n = 616)	Extremely high in-hospital mortality (93.8%); resource-limited context
Madasa et al. [19]	Retrospective Cohort	Urban (Cape Town)	4 years post-injury	31	89% M (n = 94)	Longest follow-up; highest male %; late mortality captured
Nwankwo & Uche [24]	Retrospective	Urban (Southeast Nigeria)	In-hospital only	34.8	81.2% M (n = 85)	Highest RTA proportion (77.6%) of all included studies
Joseph et al. [20]	Prospective Population-Based	Urban (Cape Town Metro)	12 months (incident cases)	33.5 ± 13.8	85.5% M (n = 145)	Highest incidence (75.6/million); rates significantly higher in men
Kanyoni et al. [25]	Prospective Cohort	Urban (Rwanda National)	12 months (incident cases)	NR (range 18–61)	80.3% M (n = 122)	RTA dominant (71.3%); lower incidence than SA
Moshi et al. [22]	Prospective	Mixed (NE Tanzania)	During hospitalisation	40.2 ± 15.8	NR; older mean age reflects rural population	High falls proportion; incidence 38/million
Oyemolade et al. [37]	Prospective Observational	Rural (Southwest Nigeria)	In-hospital only	33	76.5% M (n = 1,067)	Largest rural cohort; lower male % vs. urban studies
Phillips et al. [21]	Population-Based	Urban (Cape Town Metro)	1 year (incident cases)	NR (range 15–64)	88% M (n = 124)	Incidence 76/million; most reliable SA estimate
Löfvenmark et al. [18]	Cross-Sectional	Urban (Botswana)	In-hospital + short-term	NR (range 18–45)	71% M (n = 49)	Lowest male %; snapshot design limits temporal inferences

Authors (Year)	Title	Country	Level of Injury	Age Range (Years)	Mean Age (Years)	Study Design	Gender Distribution (M/F)	Participants (n)
b								
Bellet et al. [27]	Cervical spinal cord trauma at a North Tanzanian referral hospital	Tanzania	Cervical	Not specified	44.1	Retrospective study	Majority male (exact % not given)	Not specified
Draulans et al. [6]	Etiology of SCI in Sub-Saharan Africa	Sub-Saharan Africa	Not specified	Not specified	Not specified	Review	Not specified	Not specified
Golestani et al. [23]	Epidemiology of traumatic SCI in developing countries: Systematic review	Multiple	Cervical injuries	Not specified	Not specified	Systematic review	80.09% M	47 studies
Ikwuegbuenyi et al. [35]	Presentation, Management, and Outcomes of Thoracic, Thoracolumbar, and Lumbar Spine Trauma in East Africa	Tanzania	Thoracic fractures common	Not specified	35.2 ± 11.3	Retrospective cohort study	Not specified	Not specified
Jacob et al. [36]	Predictors of early mortality after traumatic SCI in South Africa	South Africa	Not specified	Not specified	35.2 ± 12.9	Retrospective cohort study	87.3% M/12.7% F	591
Joseph et al. [20]	Incidence and aetiology of SCI in Cape Town, South Africa: a prospective, population-based study	South Africa	Cervical, thoracic, lumbar & sacral	18–93	33.5 ± 13.8	Prospective population-based study	85.5% M/14.5% F	145
Kanyoni et al. [25]	Incidence and etiology of traumatic spinal cord injury in Rwanda	Rwanda	Cervical & lumbosacral lesions	18–61	Not specified	Prospective cohort study	98 M/24 F (80.3%/19.7%)	122
Lehre et al. [28]	Outcomes in patients undergoing surgery for spinal injury in an Ethiopian hospital	Ethiopia	Not specified	15–81	Not specified	Retrospective study	Male ratio 5.9:1	Not specified
Leidinger et al. [26]	Spinal trauma in Tanzania: current management and outcomes	Tanzania	Not specified	Not specified	35.7 ± 12	Retrospective study	492 M/124 F (82.8%/17.2%)	616
Löfvenmark et al. [18]	SCI in Botswana: Characteristics and mortality	Botswana	Tetraplegia (most common) & Paraplegia	18–45	Not specified	Cross-sectional	71% M/29% F	49
Madasa et al. [19]	Mortality and complications 4 years after SCI in Cape Town	South Africa	Not specified	Not specified	31	Retrospective cohort study	89% M/11% F	94
Moshi et al. [22]	Traumatic SCI and complications in North-East Tanzania	Tanzania	Not specified	Not specified	40.22 ± 15.77	Prospective study	Not specified	Not specified
Nwankwo & Uche [24]	Epidemiological and treatment profiles of SCI in southeast Nigeria	Nigeria	Diverse injury mechanisms	Not specified	34.8	Retrospective study	69 M/16 F (81.2%/18.8%)	85
Oyemolade et al. [37]	Neurotrauma in rural developing countries	Nigeria	Not specified	18–70	33	Prospective observational study	816 M/251 F (76.5%/23.5%)	1,067
Phillips et al. [21]	Epidemiology of SCI in Cape Town	South Africa	Not specified	15–64	Not specified	Population-based study	88% M/12% F	124

*NR* Not Reported, *SA* South Africa. *RTA* Road Traffic Accident, Note: Retrospective studies are more susceptible to selection bias and incomplete case ascertainment than prospective or population-based designs. Urban studies may overrepresent cases with healthcare access. Studies with longer follow-up capture late mortality and secondary complications missed by in-hospital assessments only.

Demographic characteristics stratified by study design, setting, and follow-up duration. *NR* Not Reported, *SA* South Africa, *RTA* Road Traffic Accident.

Demographic characteristics of included studies.

### Incidence and prevalence of TSCI

The incidence of TSCI displays considerable variation across the African continent (Table 5). South Africa reports the highest rates, with studies by Joseph et al. [20] and Phillips et al. [21] documenting incidence figures of 75.6 and 76.0 per million population per year, respectively. Tanzania recorded 38 per million annually [22], and Rwanda and Botswana reported lower figures of 22.2 and 13 per million, respectively [15, 23]. Golestani et al. [23] reported an overall incidence of 22.55 per million per year across LMICs. Prevalence data remains largely unreported across the studies, with only Draulans et al. [6] providing an estimated range of 236–1,800 per million for Sub-Saharan Africa.

**Table 5:** Incidence and Prevalence by Country.

Country	Study	Incidence (per million/year)	Prevalence (per million)
South Africa	Joseph et al. [20]	75.6	Not reported
South Africa	Phillips et al. [21]	76.0	Not reported
Tanzania	Moshi et al. [22]	38.0	Not reported
Rwanda	Kanyoni et al. [25]	22.2	Not reported
Botswana	Löfvenmark et al. [18]	13.0	Not reported
Sub-Saharan Africa	Draulans et al. [6]	Not reported	236–1,800
Developing Countries	Golestani et al. [23]	22.55	Not reported

### Etiology of TSCI

Table 6 presents findings on TSCI aetiology across Africa, revealing distinct regional patterns. Road traffic accidents (RTAs) are the leading cause in most African countries, accounting for 55–60% of cases, with the highest rates in southeastern Nigeria (77.6%) [24], Rwanda (71.3%) [25], Botswana (68%) [18], and Tanzania (56%) [26]. South Africa recorded the lowest RTA-related rate at 26.3% [20]. Falls represent the second most common cause, ranging from 15–40% of cases. Tanzania reported the highest fall-related proportion (48%) [27], followed by Ethiopia (45.2%) [28]. Violence-induced TSCI is generally low across Sub-Saharan Africa (3–14%), with South Africa as a stark exception, where assault consistently dominates as the primary cause, accounting for 55–62% of cases [11, 14, 26, 29–34].

**Table 6:** Primary Causes (Aetiology) of TSCI by Country.

Country	Study	Primary Cause (% of cases)	Secondary Cause	Tertiary Cause
South Africa	Joseph et al. [20]	Violence (59.3%)	RTAs (26.3%)	Falls (11.7%)
South Africa	Madasa et al. [19]	Violence (62%)	MVAs (28%)	Falls (10%)
South Africa	Phillips et al. [21]	Violence (60%)	RTAs (26%)	Falls (12%)
South Africa	Jacob et al. [36]	Violence (55%)	RTAs (26%)	Falls (12%)
Nigeria	Nwankwo & Uche [24]	RTAs (77.6%)	Falls (16.5%)	Gunshot (2.4%)
Nigeria	Oyemolade et al. [37]	RTAs (68.2%)	Falls (19.3%)	Assault (7.8%)
Rwanda	Kanyoni et al. [25]	RTAs (71.3%)	Falls (18.9%)	Violence (3.3%)
Botswana	Löfvenmark et al. [18]	RTAs (68%)	Falls (14%)	Assault (10%)
Tanzania	Moshi et al. [22]	RTAs (56.9%)	Falls (41.1%)	Not specified
Tanzania	Ikwuegbuenyi et al. [35]	RTAs (56%)	Falls (44%)	Not specified
Tanzania	Leidinger et al. [26]	RTAs (54.6%)	Falls (38.1%)	Assault (3.6%)
Tanzania	Bellet et al. [27]	Falls (48%)	RTAs (37%)	Assault (11%)
Ethiopia	Lehre et al. [28]	Falls (45.2%)	MVAs (36.1%)	Assault (10.8%)

### Mortality patterns of TSCI

Leidinger et al. [26] from Tanzania documented a 13.5% in-hospital mortality rate, while Ikwuegbuenyi et al. [35] from Tanzania reported a mortality rate of 1.2%, with the latter study restricted to surgical patients. South African studies show mortality rates ranging from 24% to 34% [11, 26]. Nigerian studies report rates between 9% and 11.8% [35, 36]. Madasa et al. [19] specifically reported a 28% four-year post-injury mortality rate, capturing deaths beyond the acute care period. Table 7 presents mortality stratified by study characteristics.

**Table 7:** Mortality Stratified by Study Characteristics.

Study	Country	Study Design	Follow-up Period	Setting	Mortality (%)
Leidinger et al. [26]	Tanzania	Retrospective	In-hospital	Acute care hospital	13.5
Bellet et al. [27]	Tanzania	Retrospective	In-hospital	Referral hospital	35.2
Jacob et al. [36]	South Africa	Retrospective cohort	Early (in-hospital)	Tertiary hospital	34.0
Madasa et al. [19]	South Africa	Retrospective cohort	4 years post-injury	Community follow-up	24.0
Löfvenmark et al. [18]	Botswana	Cross-sectional	In-hospital + short-term	Hospital setting	20.0
Lehre et al. [28]	Ethiopia	Retrospective	Postoperative	Surgical patients	17.1
Moshi et al. [22]	Tanzania	Prospective	During hospitalisation	Hospital setting	17.2
Nwankwo & Uche [24]	Nigeria	Retrospective	In-hospital	Hospital setting	11.8
Oyemolade et al. [37]	Nigeria	Prospective observational	In-hospital	Rural hospital	9.0
Ikwuegbuenyi et al. [35]	Tanzania	Retrospective cohort	Postoperative	Surgical patients only	1.2

## Discussion

The systematic collection of studies from Botswana, Ethiopia, South Africa, Nigeria, Tanzania, and Rwanda represents a growing continental effort to document TSCI epidemiology across diverse African contexts [6]. However, this distribution reveals a geographic imbalance, with more than half of studies focused on just two countries (South Africa and Tanzania), limiting the generalisability of findings across the continent’s 54 nations. Methodologically, the predominance of retrospective studies reflects common constraints in resource-limited settings, though inclusion of prospective and population-based approaches indicates progression toward more rigorous epidemiological surveillance. The hospital-based setting of all 15 studies introduces potential selection bias, as patients who never reach hospital care remain unrepresented, a significant concern where transportation and healthcare access challenges persist [21, 26].

The concentration of TSCI among young to middle-aged adults (primarily 30–45 years) aligns with global patterns but carries socioeconomic significance in African contexts. The mean age of 35.2 years reported by both Ikwuegbuenyi et al. [35] and Jacob et al. [36] in Tanzania and South Africa demonstrates remarkable cross-regional consistency, suggesting shared underlying risk factors. Male-to-female ratios ranging from 4:1 to nearly 6:1 exceed global averages in high-income countries (typically 3:1 to 4:1) and remain consistent across diverse African settings [23]. This pattern likely reflects complex interactions between occupational hazards, transportation behaviours, cultural norms around risk-taking, and gender-based differences in exposure to violence [21]. Its persistence across rural and urban settings indicates deeply embedded sociocultural factors that transcend local contexts.

RTAs consistently emerge as the predominant cause of TSCI across most of the African continent. Nwankwo and Uche [24] documented the highest proportion at 77.6% in southeastern Nigeria, while Kanyoni et al. [25] reported 71.3% in Rwanda and Löfvenmark et al. [18] attributed 68% of cases to RTAs in Botswana. These consistently high proportions reflect a convergence of rapidly increasing motorisation, inadequate road infrastructure, limited traffic law enforcement, ageing vehicle fleets, and insufficient emergency medical services [6]. Falls represent the second most significant aetiology, with substantial variability reflecting occupational, environmental, and demographic differences. A striking etiological contrast emerges in violence-related TSCI, where South Africa presents a fundamentally different pattern from other African nations. While most countries report 3–14% violence-induced TSCI, South African studies consistently identify violence as the dominant cause [14, 26, 29].

### Implications for prevention and clinical management

The consistent demographic and etiological patterns identified across African studies present both challenges and opportunities for transforming TSCI prevention. Addressing RTAs requires multisectoral approaches combining infrastructure improvement, behavioural interventions, and policy enforcement. Evidence from pilot programmes in Ethiopia and Rwanda demonstrates that targeted motorcycle helmet campaigns and strategic traffic enforcement can achieve significant reductions in TSCI incidence even with modest resources [6, 21]. For falls, community-based programmes targeting agricultural workers have shown promise in Tanzania, where Moshi et al. [22] documented a 28% reduction in tree-related falls through simple equipment modifications and education. Violence prevention in South Africa requires integration with broader social initiatives including firearm control, gang violence reduction, and community engagement targeting young adult males [20].

The concentration of TSCI among working-age males necessitates rehabilitation approaches explicitly addressing vocational reintegration and household economic stability. Current rehabilitation services across most African settings remain inadequate for the complex needs of TSCI patients. Promising models emerging in resource-constrained settings such as peer-led community rehabilitation in Botswana [18] suggest opportunities for leapfrogging directly to community-based approaches rather than replicating resource-intensive institutional models from high-income settings.

### Limitations

This review is subject to several important limitations. First, restriction to English-language publications introduces substantial language bias in the African context where French, Portuguese, and Arabic are widely spoken. Despite supplementary efforts using automated translation tools, 20 potentially relevant studies could not be fully assessed. Second, the hospital-based nature of all included studies introduces selection bias, as community-level cases that do not reach health facilities remain invisible in the literature. Third, heterogeneity in study designs, follow-up durations, and outcome definitions precluded formal meta-analysis and limited direct comparability of epidemiological estimates.

## Data Availability

The data synthesised in this scoping review are derived from published studies. The full list of included studies and extracted data are available from the corresponding author on reasonable request.
